# Contraction-Induced Loss of Plasmalemmal Electrophysiological Function Is Dependent on the Dystrophin Glycoprotein Complex

**DOI:** 10.3389/fphys.2021.757121

**Published:** 2021-10-26

**Authors:** Cory W. Baumann, Angus Lindsay, Sylvia R. Sidky, James M. Ervasti, Gordon L. Warren, Dawn A. Lowe

**Affiliations:** ^1^Department of Biomedical Sciences, Ohio Musculoskeletal and Neurological Institute (OMNI), Ohio University, Athens, OH, United States; ^2^Divisions of Rehabilitation Science and Physical Therapy, Department of Rehabilitation Medicine, University of Minnesota, Minneapolis, MN, United States; ^3^School of Exercise and Nutrition Sciences, Institute for Physical Activity and Nutrition, Deakin University, Geelong, VIC, Australia; ^4^Department of Biochemistry, Molecular Biology and Biophysics, University of Minnesota, Minneapolis, MN, United States; ^5^Department of Physical Therapy, Georgia State University, Atlanta, GA, United States

**Keywords:** eccentric contractions, electromyography, injury, muscular dystrophy, strength

## Abstract

Weakness and atrophy are key features of Duchenne muscular dystrophy (DMD). Dystrophin is one of the many proteins within the dystrophin glycoprotein complex (DGC) that maintains plasmalemmal integrity and cellular homeostasis. The dystrophin-deficient *mdx* mouse is also predisposed to weakness, particularly when subjected to eccentric (ECC) contractions due to electrophysiological dysfunction of the plasmalemma. Here, we determined if maintenance of plasmalemmal excitability during and after a bout of ECC contractions is dependent on intact and functional DGCs rather than, solely, dystrophin expression. Wild-type (WT) and dystrophic mice (*mdx*, mL172H and *Sgcb^−/−^* mimicking Duchenne, Becker and Limb-girdle Type 2E muscular dystrophies, respectively) with varying levels of dystrophin and DGC functionality performed 50 maximal ECC contractions with simultaneous torque and electromyographic measurements (M-wave root-mean-square, M-wave RMS). ECC contractions caused all mouse lines to lose torque (*p*<0.001); however, deficits were greater in dystrophic mouse lines compared to WT mice (*p*<0.001). Loss of ECC torque did not correspond to a reduction in M-wave RMS in WT mice (*p*=0.080), while deficits in M-wave RMS exceeded 50% in all dystrophic mouse lines (*p*≤0.007). Moreover, reductions in ECC torque and M-wave RMS were greater in *mdx* mice compared to mL172H mice (*p*≤0.042). No differences were observed between *mdx* and *Sgcb^−/−^* mice (*p*≥0.337). Regression analysis revealed ≥98% of the variance in ECC torque loss could be explained by the variance in M-wave RMS in dystrophic mouse lines (*p*<0.001) but not within WT mice (*R*^2^=0.211; *p*=0.155). By comparing mouse lines that had varying amounts and functionality of dystrophin and other DGC proteins, we observed that (1) when all DGCs are intact, plasmalemmal action potential generation and conduction is maintained, (2) deficiency of the DGC protein β-sarcoglycan is as disruptive to plasmalemmal excitability as is dystrophin deficiency and, (3) some functionally intact DGCs are better than none. Our results highlight the significant role of the DGC plays in maintaining plasmalemmal excitability and that a collective synergism (*via* each DGC protein) is required for this complex to function properly during ECC contractions.

## Introduction

Duchenne muscular dystrophy (DMD), one of the most serious genetic childhood diseases, is characterized by progressive weakness and atrophy after repetitive cycles of degeneration and regeneration due to the lack of dystrophin (Bell and Conen, 1968; Kinali et al., 2011; Vohra et al., 2015). The dystrophin-deficient *mdx* mouse also suffers from repetitive muscle degeneration (Pastoret and Sebille, 1995), which is one of the reasons why it has been extensively used as a model of human DMD. A key feature of *mdx* mice is that muscles composed of predominantly fast-twitch fibers are hypersensitive to losing strength in response to eccentric (ECC) contractions (Head et al., 1992; Moens et al., 1993; Lindsay et al., 2019; Kiriaev et al., 2021). Numerous laboratories have used *ex vivo*, *in situ*, or *in vivo* muscle preparations to demonstrate that loss of strength during and immediately following ECC contractions is 20–60% greater in *mdx* compared with wild-type (WT) mice (Moens et al., 1993; Petrof et al., 1993; Brooks, 1998; Call et al., 2011; Pratt et al., 2013, 2015; Baumann et al., 2020; Lindsay et al., 2020). Susceptibility to ECC contraction-induced strength loss in *mdx* muscles has therefore become a standard outcome measure in preclinical studies to assess disease severity and the efficacy of potential therapies for DMD. However, despite the widespread use of ECC contraction protocols, the muscular dystrophy field remains uncertain what underlying mechanism initiates the immediate strength loss in *mdx* muscle. Function of several cellular and subcellular structures in muscle that are necessary for contraction and force generation have been reported to be disrupted following ECC contractions in *mdx* muscle, including the neuromuscular junction (NMJ) (Pratt et al., 2013, 2015), plasmalemma (Petrof et al., 1993; Call et al., 2013; Roy et al., 2016), ryanodine receptor (Bellinger et al., 2009; Lindsay et al., 2020), sarco/endoplasmic reticulum Ca^2+^-ATPase (Mázala et al., 2015; Lindsay et al., 2020), and myofibrillar proteins (Blaauw et al., 2010).

All the aforementioned structures are putative sites contributing to contraction-induced strength loss in *mdx* muscle (see review by Warren et al. (2001) for WT muscle). However, we posit that strength loss in *mdx* muscle is initially triggered, in large, by plasmalemmal inexcitability (Call et al., 2013; Baumann et al., 2020). Three main arguments can be made to support this proposition. First, it has repeatedly been demonstrated that *in vivo* ECC contraction-induced reductions in peak torque in *mdx* muscle parallel reductions of M-wave root-mean-square (M-wave RMS; i.e., a measure of plasmalemmal excitability; Call et al., 2013; Roy et al., 2016; Baumann et al., 2020). Second, loss of M-wave RMS corresponds with the resting membrane potential of fibers becoming more positive (i.e., depolarized; Call et al., 2013). Finally, M-wave RMS and the resting membrane potential in WT muscle fibers following ECC contractions are unchanged or only marginally decreased (Warren et al., 1999; Call et al., 2013; Baumann et al., 2020). These three points suggest ECC contractions depolarize *mdx* fibers leaving them inexcitable (i.e., unable to generate and/or conduct action potentials). Fewer action potentials will be observed as reductions in M-wave RMS and, inevitability, the muscle’s force generating capacity (Call et al., 2013; Baumann et al., 2020). Surprisingly, despite peak isometric torque and M-wave RMS being reduced approximately 40–60% after a single bout of 50–100 maximal ECC contractions, *mdx* muscle exhibits a remarkable ability to recover, as both variables return to baseline within 2weeks (Call et al., 2013; Baumann et al., 2020). These results indicate dystrophin may be necessary for excitation to occur at the plasmalemma during a series of ECC contractions, but not essential for complete recovery of plasmalemma electrophysiological function or isometric strength (Call et al., 2013; Baumann et al., 2020).

Dystrophin is one of the many proteins that make up the dystrophin glycoprotein complex (DGC). The DGC is also composed of cytoplasmic, transmembrane, and extracellular proteins including the sarcoglycans, dystroglycans, dystrobrevins, syntrophins, sarcospan, caveolin-3, and neuronal nitric oxide (NO) synthase (Straub and Campbell, 1997; Lapidos et al., 2004). It has become increasingly clear that the DGC holds both structural and signal transduction properties (Lapidos et al., 2004; Constantin, 2014; Garbincius and Michele, 2015) and provides a strong mechanical link from the intracellular cytoskeleton to the extracellular matrix (Rybakova et al., 2000). In our most recent publication, we suggested that future work will need to determine how DGC proteins, in addition to dystrophin, contribute to electrophysiological (dys)function of the plasmalemma (Baumann et al., 2020).

To advance on our suggested work, here, we strategically selected four different mouse lines that possess varying contents of dystrophin and DGC proteins as well as functionality. WT mice served as healthy controls while *mdx*, mL172H and *Sgcb^−/−^* mice represented lines mimicking Duchenne, Becker and Limb-girdle Type 2E muscular dystrophies, respectively (Ng et al., 2012; McCourt et al., 2018). As mentioned, *mdx* mice are not only dystrophin deficient but also have reduced contents of other DGC proteins (Ohlendieck and Campbell, 1991). The transgenic mL172H mouse expresses dystrophin with an L172H missense mutation associated with Becker muscular dystrophy on the *mdx* background (McCourt et al., 2018). The mutant L172H dystrophin is expressed at ~40% of WT and localizes to the plasmalemma but only partially restores the expression of other DGC components (McCourt et al., 2018). In contrast, the *Sgcb^−/−^* mouse expresses WT levels of dystrophin at the plasmalemma and retains the DGC but lacks the entire sarcoglycan complex due to the absence of β-sarcoglycan (Araishi et al., 1999; Durbeej et al., 2000). Using these mouse lines, which retain varying degrees of dystrophin and DGC protein contents and functionality, we hypothesized that in order to attenuate ECC contraction-induced plasmalemmal inexcitability, dystrophin would not only need to be present but also “functional” (Baumann et al., 2020). Stated differently, maintaining electrophysiological function of the plasmalemma would not solely be dependent on having dystrophin but rather having dystrophin working collectively with other DGC proteins. Therefore, the purpose was to determine if maintenance of plasmalemmal excitability was dependent on completely intact and fully functional DGCs. To accomplish this, we measured M-wave RMS and torque before, during and after a single bout of ECC contractions in WT, *mdx*, mL172H, and *Sgcb^−/−^* mice.

## Materials and Methods

### Ethical Approval and Animal Models

WT (C57BL/10) and *mdx* (C57BL/10-DMD^mdx^) mice were obtained from Jackson Laboratory (Bar Harbor, ME) or bred locally from these mice. *Sgcb^−/−^* and mL172H-*mdx* mice were generated as previously described (Belanto et al., 2016; McCourt et al., 2018) and bred locally. To note, *Sgcb^−/−^* mice were originally obtained from Jackson Laboratories (B6.129-*^Sgcbtm1Kcam^/*1J) and backcrossed onto the C57BL/6 strain. All other groups were on a C57BL/10 background. Mice were male and 4–8months of age. This age range was selected to avoid the variability associated with the peak cycles of degeneration and regeneration observed in young *mdx* mice, because the primary objective of this research was to investigate the mechanisms of immediate strength loss in dystrophic muscle, rather than the mechanisms of disease onset or pathology. Indeed, at 3weeks of age, *mdx* muscles undergo cycles of myonecrosis and concurrent regeneration that results in nearly every myofiber being repaired by 6–12weeks of age (Duddy et al., 2015; Massopust et al., 2020). All mice were fed with food and water *ad libitum* on a 14/10h light/dark cycle. Animal procedures were in accordance with the standards set by the Institutional Animal Care and Use Committees at the University of Minnesota.

### Experimental Design

To test the hypothesis that completely intact and fully functional DGCs are necessary to attenuate ECC contraction-induced plasmalemma inexcitability, *in vivo* torque and M-wave RMS were recorded before, during, and after a single bout of ECC contractions. Briefly, the anterior crural muscles [TA, extensor digitorum longus (EDL), and extensor hallucis muscles] were studied in all groups using *in vivo* physiology. To activate the anterior crural muscle, the left common peroneal nerve was stimulated using percutaneous needle electrodes or *via* a chronically implanted nerve cuff to activate the anterior crural muscles. Anterior crural muscle isometric torque was then measured before and immediately after a single bout of 50 maximal ECC contractions. To determine whether plasmalemmal electrophysiological function was impaired by the ECC contractions, similar to what we have previously shown in *mdx* muscle (Call et al., 2013; Baumann et al., 2020), mice with nerve cuffs were also implanted with EMG electrodes around left TA muscle. Plasmalemmal electrophysiological function, measured by M-wave RMS, was recorded simultaneously with each muscle contraction. After the final contraction, mice were euthanized and the TA muscles were dissected, flash frozen, stored at −80°C, and later used for immunoblotting.

It is important to note that anterior crural muscles of *mdx* mice are considered as fast-twitch. Specifically, fibers of TA and EDL muscles express primarily the type IIb myosin heavy chain isoform (Schertzer et al., 2006; Landisch et al., 2008; Péladeau et al., 2018). Therefore, because fast-twitch *mdx* muscles appear to be the most hypersensitive to ECC contraction-induced strength loss (Head et al., 1992; Moens et al., 1993; Lindsay et al., 2019), the conclusions drawn from this study may not be directly translatable to *mdx* muscles such as the soleus that express predominately type I and IIa myosin heavy chain isoforms (Landisch et al., 2008). For additional information on ECC contraction-induced strength loss across muscle fiber types in *mdx* mice, readers are directed to Lindsay et al. (2019) and Kiriaev et al. (2021).

### Experimental Methodology

#### Anesthesia and Euthanasia

For surgeries (implantation of nerve cuff and EMG electrodes) and physiological experiments (torque and EMG measurements), mice were initially anesthetized in an induction chamber using isoflurane and then maintained by the inhalation of ~1.5% isoflurane mixed with oxygen at a flow rate of 125ml·min^−1^. This anesthetic regimen was also used when torque and EMG measurements were made. After the final contraction protocol, mice were euthanized with an overdose of sodium pentobarbital (150mg·kg^−1^ body mass) or cervically dislocated while under anesthesia.

#### Surgical Procedures

An incision was made through the biceps femoris muscle in the left hindlimb, and a nerve cuff made of platinum iridium wire (Medwire-Sigmund Chon 10Ir9/49T, Mt. Vernon, NY) and silastic tubing was placed around the common peroneal nerve (Warren et al., 1998, 1999, 2000; Baumann et al., 2020). No less than 14days after implanting the stimulating nerve cuff, TA muscle EMG electrodes to record M-waves were then implanted in the anesthetized mouse (Warren et al., 1999, 2000; Baumann et al., 2020). Briefly, deinsulated ends of two platinum iridium wires, offset by ~2mm, were routed underneath the superficial fascial sheath of the TA muscle. The electrode wire spacing theoretically permitted sampling of EMG activity from the full thickness of the TA muscle beneath the electrodes (Basmajian and De Luca, 1985; Warren et al., 1999). The wires were secured to adjacent tissue, and the proximal ends of the wires were run subcutaneously to the dorsal cervical region and connected to an EMG amplifier (Model P55, Grass Technologies). *In vivo* muscle testing with simultaneous M-wave measurements was initiated no less than 14days after the EMG wire implantation. We have previously shown that these techniques are highly reliable and reproducible across time and laboratories (Warren et al., 1998, 1999, 2000; Call et al., 2013; Baumann et al., 2020) and therefore, only a subset of mice (6–10 per strain) underwent both surgeries.

#### Torque Measurements

*In vivo* isometric torque of the anterior crural muscles was assessed as previously described (Lowe et al., 1995; Baumann et al., 2014, 2020). The anesthetized mouse (see section Ethical Approval and Animal Models) was placed on a temperature-controlled platform to maintain core temperature at 37°C, and the left knee was clamped and the left foot was secured to an aluminum footplate that is attached to the shaft of the servomotor system (Model 300B-LR; Aurora Scientific, Aurora, Ontario, Canada). The proximal end of the nerve cuff, which was run subcutaneously to the dorsal cervical region, was then connected to a stimulator and stimulus isolation unit (Models S48 and SIU5, respectively; Grass Technologies, West Warwick, RI). For mice without nerve cuffs, sterilized platinum needle electrodes were precisely inserted through the skin for stimulation of the left common peroneal nerve and connected to the stimulator and stimulus isolation unit. The contractile function of the anterior crural muscles was assessed by measuring isometric torque as a function of stimulation frequency (torque frequency protocol; 20–300Hz; 150-ms train with 0.1-ms pulses). Peak isometric torque was recorded as the highest tetanic torque obtained during the torque-frequency protocol.

#### Injury Protocol

Approximately 1–2min after the torque-frequency protocol, the anterior crural muscles were injured by performing electrically stimulated maximal ECC contractions (Lowe et al., 1995; Baumann et al., 2020; Sidky et al., 2021). During each ECC contraction, the foot was passively moved from 0° (positioned perpendicular to tibia) to 19° of dorsiflexion, where the anterior crural muscles performed a 100-ms isometric contraction followed by an additional 20ms of stimulation while the foot was moved to 19° of plantarflexion at 2,000°·s^−1^. A 5-min rest after the ECC contraction protocol was given before reassessing contractile function *via* the torque-frequency protocol.

#### M-wave Assessment

Analysis of the electrically evoked myoelectric signal was done using the amplitude measure (i.e., M-wave RMS) as previously described (Warren et al., 1999, 2000; Baumann et al., 2020). M-wave RMS was calculated for the full 150ms of the isometric contractions during the torque-frequency protocols and across the first 100ms (i.e., the isometric portion) of the ECC contraction protocol. Because the anterior crural muscles were maximally recruited *via* electrical stimulation of the common peroneal nerve, a decrease in M-wave RMS was interpreted as impairment of action potential generation and/or conduction (Warren et al., 1999; Call et al., 2013).

#### Immunoblots

The proximal half TA muscles were homogenized in an ice-cold lysis and extraction buffer of the following constituents (in mmol L^−1^): 250 sucrose, 100 KCl, 20 MOPS, and 5 EDTA (pH 6.8). The buffer was supplemented with 100x protease/phosphatase inhibitor cocktail (Thermo Scientific, Rockford, IL). Total protein content was quantified using the A280 method on a NanoDrop spectrophotometer. Equal amounts of protein (30μg) were loaded onto 4–15% SDS polyacrylamide gel and separated according to molecular weight (150V for 45min). The proteins were then transferred to a PVDF membrane using a wet transfer system at 110V for 90min (Bio-Rad Laboratories, Hercules, CA) and blocked for 60min at room temperature in 5% non-fat dried milk (w/v) dissolved in tris-buffered saline with 0.1% Tween-20 (TBS-T). Membranes were then probed with anti-dystrophin (1:1,000; Novus, NBP2-66815), β-sarcoglycan (1:1,000; Novus, NBP1-90300), and GAPDH (1:5,000; Cell Signaling, D4C6R) primary antibodies overnight in a cold room (4°C) on an orbital shaker. Following incubation in the primary antibody, membranes were washed with TBS-T (3×5min) then probed with secondary IgG Dylight antibodies (1:10,000; Cell Signaling, 5,470 and 5,366) for 60min at room temperature on an orbital shaker. The membranes were then washed as described above and the secondary antibody signal was visualized on LI-COR’s Odyssey Infrared Imaging System. Band density was calculated with Odyssey software v2.1. Dystrophin and β-sarcoglycan content were normalized to GAPDH.

### Statistical Analyses

A one-way ANOVA was used to assess differences for peak isometric and ECC torques and M-wave RMS across mouse lines followed by Tukey *post hoc* tests when significance was detected. When comparing peak isometric and ECC torque and M-wave RMS within the same mouse, a paired-samples *t* test was utilized. Differences in protein content between mouse lines were determined using a Mann–Whitney test. Linear regression was used to assess the relationship between torque and M-wave RMS. Significance was set at *p*<0.05. Values are expressed as mean±SD. All statistical testing was performed using Prism 9 software (GraphPad, San Diego, CA).

## Results

### Protein Contents

**Figure 1:**
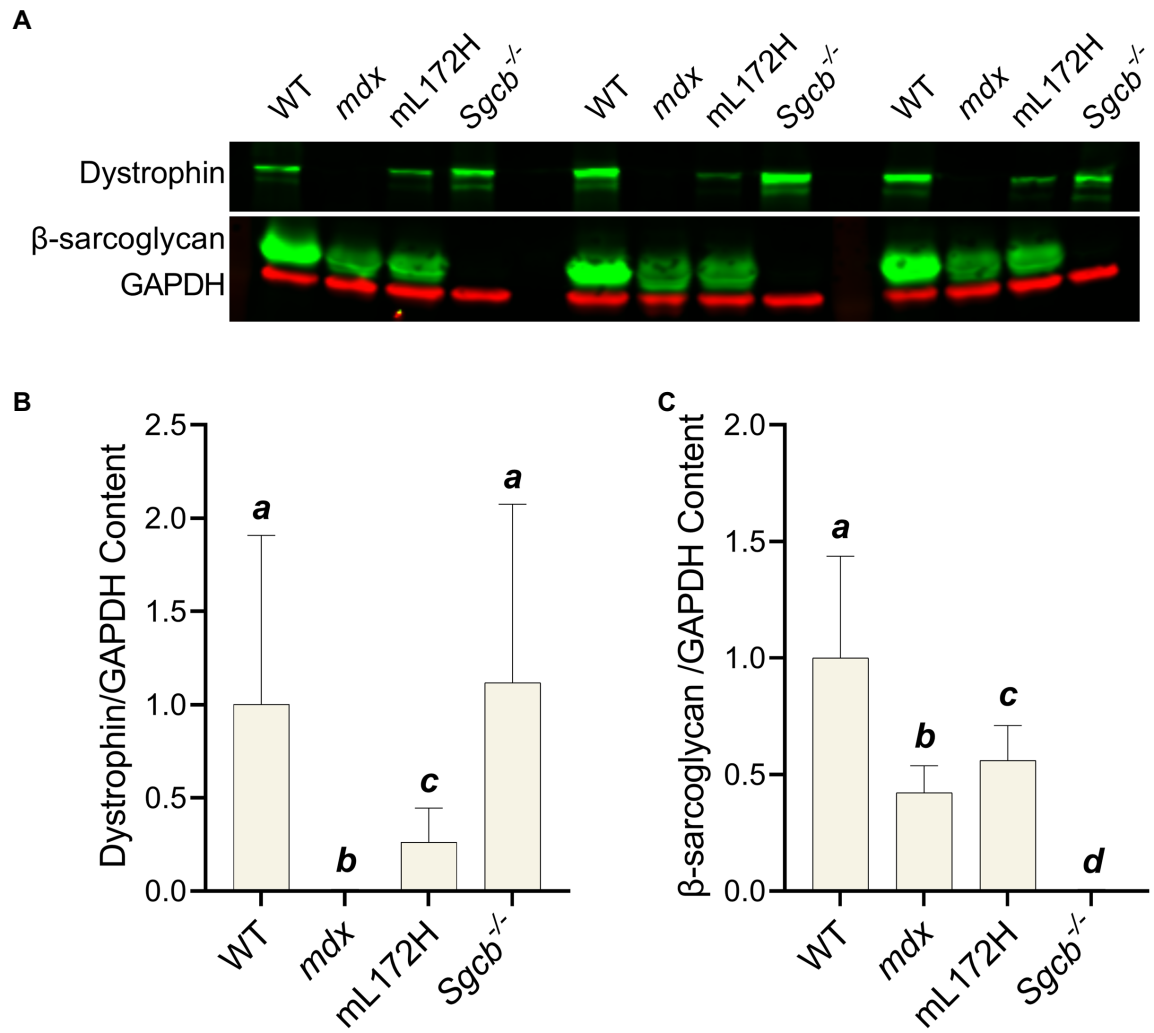
Immunoblots confirm dystrophic mouse lines. **(A)** Representative immunoblots of three individual mice per strain. A blank lane separates *Sgcb^−/−^* and WT between sets. **(B)** Dystrophin content normalized to GAPDH. **(C)** β-sarcoglycan content normalized to GAPDH. Values are normalized to WT and set to 1.0. Sample size is nine mice per group. Groups with the *same letter* are not significantly different from each other. Significance was set at *p*<0.05. Bars are mean±SD.

Immunoblotting was performed on TA muscles to confirm dystrophic mouse lines. Representative blots of dystrophin, β-sarcoglycan, and GAPDH from muscle of three individual mice per line are depicted in Figure 1A. *Mdx* (Figure 1B) and *Sgcb^−/−^* (Figure 1C) muscle did not contain dystrophin and β-sarcoglycan (*p*<0.001), respectively. Dystrophin (albeit a mutated form of dystrophin) from mL172H muscle was 26% of that observed in WT muscle (*p*=0.024). Dystrophin content did not differ between WT and *Sgcb^−/−^* muscle (Figure 1B; *p*=0.863). β-sarcoglycan content was lower in *mdx* and mL172H muscle compared to WT muscle (*p*≤0.004), while *mdx* muscle had less β-sarcoglycan than that of mL172H muscle (Figure 1C; *p*=0.024).

### Peak Isometric and ECC Torque

**Figure 2:**
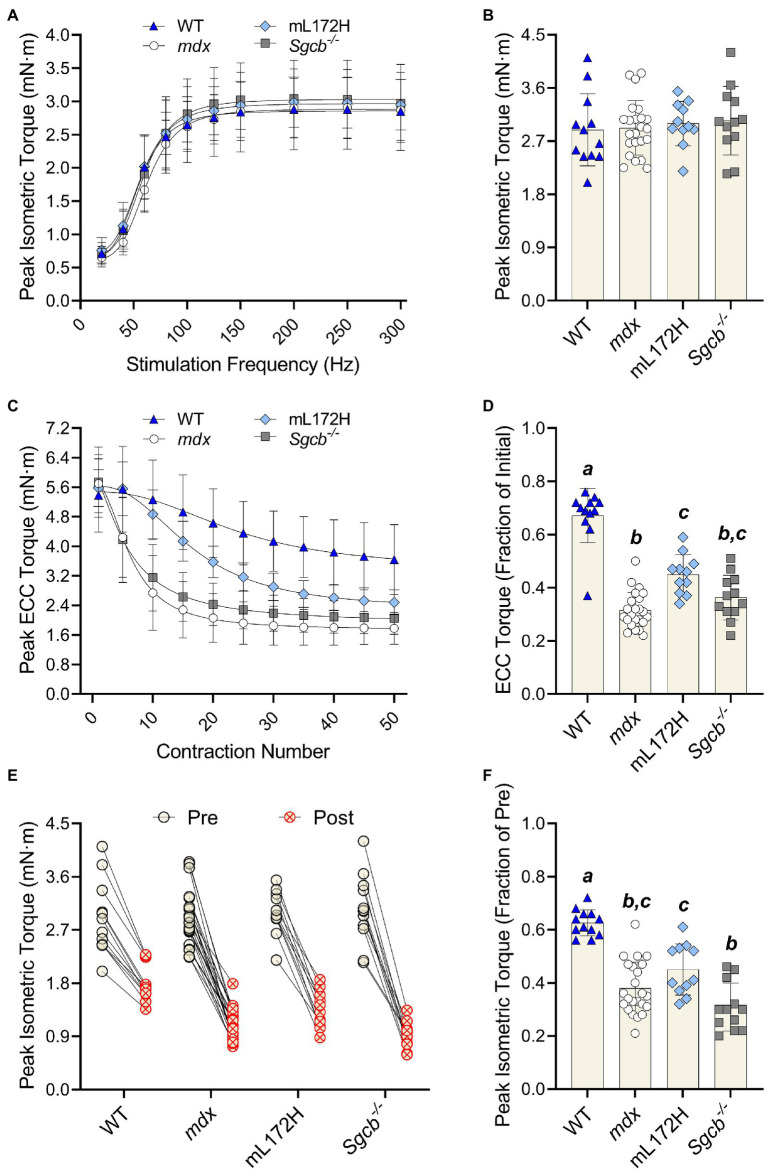
Dystrophic mouse lines are hypertensive to ECC contraction-induced strength loss. **(A)** Pre-injury torque-frequency curves. Peak isometric torque was characterized as the highest tetanic contraction obtained during the torque-frequency protocol while **(B)** depicts pre-injury peak isometric torque. **(C)** Peak ECC torque over 50 maximal contractions. **(D)** ECC torque deficits expressed as a fractional change from the first to fiftieth contraction. **(E)** Peak isometric torque of each individual mouse pre- and post-ECC contractions; solid line between Pre and Post indicates same mouse. **(F)** Peak isometric torque expressed as a fractional change pre- to post-ECC contractions. Groups with the *same letter* are not significantly different from each. Significance was set at *p*<0.05. Bars are mean±SD.

The highest torque obtained during the torque-frequency protocol was defined as peak isometric torque (Figure 2A). Despite *mdx*, mL172H and *Sgcb^−/−^* mice being considered dystrophic mouse lines, peak dorsiflexor torque did not differ between lines or from WT mice (Figure 2B; *p*=0.861). As with peak isometric torque, ECC torque produced during the first ECC contraction did not differ across groups (Figure 2C; *p*=0.678). The performance of 50 maximal ECC contractions caused all mouse lines to lose torque (*p*<0.001). However, across groups, ECC torque deficits were greater in the dystrophic mouse lines compared to WT mice (Figures 2C,D; *p*<0.001). Moreover, ECC torque deficits were greater in *mdx* mice compared to mL172H mice (*p*<0.001), yet similar between mL172H and *Sgcb^−/−^* mice (*p*=0.059). No differences were observed between *mdx* and *Sgcb^−/−^* mice (Figure 2D; *p*=0.337).

The performance of 50 maximal ECC contractions reduced isometric torque in all mouse lines (Figure 2E; *p*<0.001). As with ECC torque, isometric torque deficits were greater (pre- to post-ECC contractions) among the dystrophic mouse lines compared to that of WT mice (Figure 2F; *p*≤0.001). Contrary to the mL172H mice showing some protection during the ECC contraction protocol, post-ECC isometric torque deficits were not different between mL172H and *mdx* mice (*p*=0.141). *Sgcb^−/−^* mice did not differ from *mdx* mice (*p*=0.125; Belanto et al., 2016), yet experienced greater isometric torque deficits than mL172H mice (Figure 2F; *p*=0.002).

### Peak Isometric and ECC M-wave RMS

**Figure 3:**
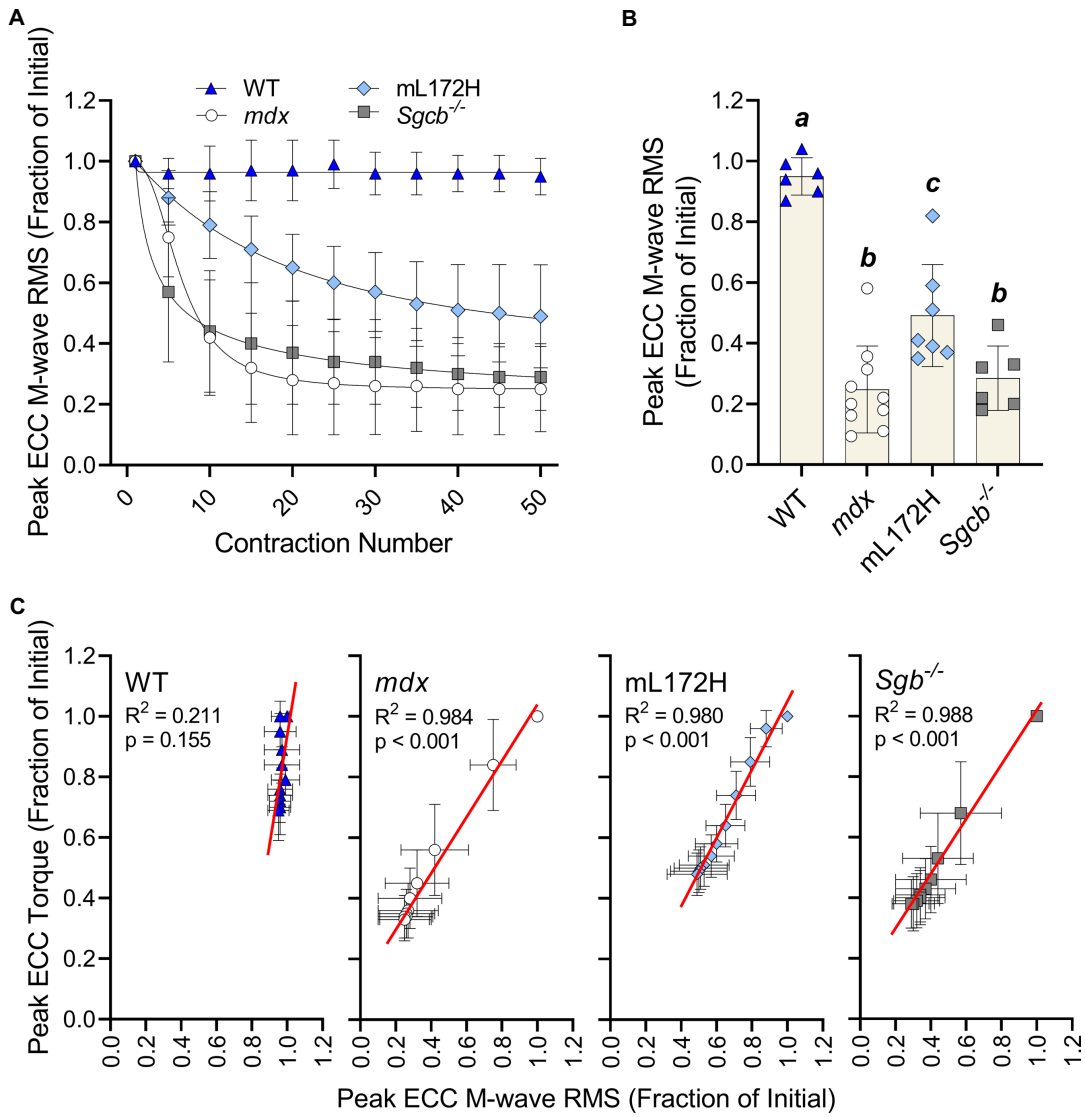
Loss of M-wave RMS parallels loss of torque in dystrophic mouse lines. **(A)** M-wave RMS in WT, *mdx*, mL172H, and *Sgcb^−/−^* mice over 50 maximal ECC contractions. **(B)** Peak ECC M-wave RMS expressed as a fractional change from the first to fiftieth contraction. **(C)** Regression analysis of peak ECC torque and M-wave RMS in WT and dystrophic mouse lines. Groups with the *same letter* are not significantly different from each. Significance was set at *p*<0.05. Bars are mean±SD.

Figure 3A depicts M-wave RMS deficits over the ECC contraction protocol. When comparing the M-wave RMS recorded during the first to fiftieth ECC contraction, no change was observed in WT mice (*p*=0.080), while all dystrophic mouse lines decreased over 50% (Figures 3A,B; *p*≤0.007). However, deficits in M-wave RMS were greater in *mdx* and *Sgcb^−/−^* mice than that recorded in mL172H mice (*p*≤0.042). *Mdx* and *Sgcb^−/−^* mice did not differ (Figure 3B; *p*=0.945). Regression analysis revealed that ≥98% of the variance in ECC torque loss could be explained by the variance in M-wave RMS in the dystrophic mouse lines (Figure 3C; *p*<0.001), while in WT mice, none of the variance in ECC torque could be explained statistically by the variance in M-wave RMS (*R*^2^=0.211; *p*=0.155).

**Figure 4:**
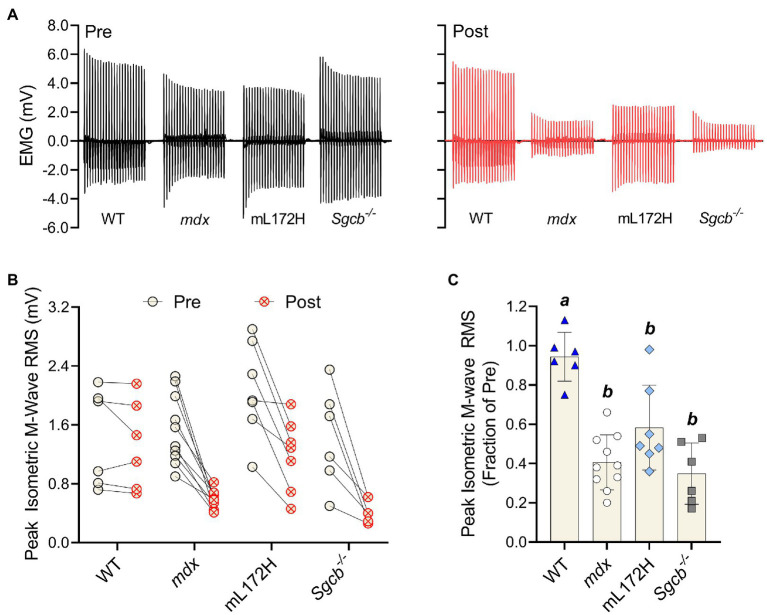
Dystrophin content does not prevent loss of plasmalemmal excitability following a bout of ECC contractions. **(A)** Representative peak isometric EMG tracings pre- and post-ECC contractions. **(B)** Peak isometric M-wave RMS of each individual mouse pre- and post-ECC contractions; solid line between Pre and Post indicates same mouse. **(C)** Peak isometric M-wave RMS expressed as a fractional change pre- to post-ECC contractions. Groups with the *same letter* are not significantly different from each. Significance was set at *p*<0.05. Bars are mean±SD.

M-wave RMS was also recorded simultaneously with isometric torque measures; representative tracings are presented in Figure 4A. When comparing pre- to post-ECC contractions, M-wave RMS did not differ in WT mice (*p*=0.311) yet decreased over 40% in all dystrophic mouse lines (Figures 4B,C; *p*≤0.011). Deficits in M-wave RMS were greater in all dystrophic mouse lines when compared to WT mice (*p*≤0.003), yet did not differ from one another (i.e., mL172H, *mdx* and *Sgcb^−/−^* mice; Figure 4C; *p*≥0.068). Changes in isometric torque and M-wave RMS from pre- to post-injury were reflective of those during the ECC contractions.

## Discussion

The purpose of this study was to determine if loss of plasmalemmal excitability is dependent on having completely intact and fully functional DGCs rather than just dystrophin content. By comparing mouse lines that had varying amounts and functionality of dystrophin and other DGC proteins, we observed three primary findings. First, M-wave RMS does not change during or after a bout of ECC contractions in WT mice, meaning that when all DGCs are intact, plasmalemmal action potential generation and conduction are maintained. Second, *Sgcb^−/−^* mice lose M-wave RMS due to ECC contractions, which parallels that observed in *mdx* mice, indicating that deficiency of the DGC protein β-sarcoglycan is as disruptive to plasmalemmal excitability as is dystrophin deficiency. Third, mL172H mice are also susceptible to ECC-induced reductions in M-wave RMS yet show marginal protection when compared to *mdx* or *Sgcb^−/−^* mice, suggesting some functionally intact DGCs are better than none. These results support our hypothesis that maintaining electrophysiological function of the plasmalemma during ECC contractions is dependent on dystrophin working collectively with other DGC proteins.

*Mdx*, mL172H, and *Sgcb^−/−^* mouse lines model Duchenne, Becker, and Limb-girdle Type 2E muscular dystrophies, respectively. Importantly, we specifically selected these three dystrophic mouse lines to assess how altered expression level and functionality of dystrophin and the DGC impacts plasmalemmal excitability during and after ECC contractions. *Mdx* mice are dystrophin deficient and have reduced contents of other DGC proteins (Ohlendieck and Campbell, 1991). For instance, we found that β-sarcoglycan was 58% lower in *mdx* muscle compared to that of WT muscle. *Sgcb^−/−^* mice are β-sarcoglycan deficient and lack the entire sarcoglycan complex, but express WT levels of plasmalemmal dystrophin and DGCs (Araishi et al., 1999; Durbeej et al., 2000). The mL172H mouse expresses a mutated form of dystrophin that is approximately 30–40% of WT (present study; McCourt et al., 2018). However, expression of L172H dystrophin does not completely restore other DGC components to WT levels yet properly localizes DGC components to the plasmalemma (McCourt et al., 2018). Regardless of how dystrophin or the DGC was perturbed, all dystrophic lines (*mdx*, mL172H, and *Sgcb^−/−^*) were hypersensitive to ECC contraction-induced strength loss when compared to WT mice. We posit that these strength deficits were due to reductions in plasmalemmal excitability that stemmed from only partially complete DGCs or complete lack thereof.

Various cytoplasmic, transmembrane, and extracellular proteins make up the DGC including dystrophin, sarcoglycans, dystroglycans, dystrobrevins, syntrophins, sarcospan, caveolin-3, and neuronal nitric oxide (NO) synthase (Straub and Campbell, 1997; Lapidos et al., 2004). Functionally, the DGC provides a strong mechanical link from the intracellular cytoskeleton to the extracellular matrix (Rybakova et al., 2000) and holds both structural and signal transduction properties (Lapidos et al., 2004; Constantin, 2014; Garbincius and Michele, 2015) making it hard to discern how the DGC specifically influences plasmalemmal excitability. Loss of M-wave RMS could be due to transmission failure at the NMJ or an impaired ability to generate and/or conduct an action potential along the plasmalemma. NMJ transmission failure is largely based on histological evidence demonstrating that NMJ morphology is altered after performing ECC contractions in *mdx* mice but maintained in WT mice (Pratt et al., 2013, 2015). Conversely, loss of M-wave RMS due to plasmalemmal dysfunction is based on data obtained from *mdx* muscle fibers, in which approximately 50% were more positive than −55mV compared with only 7% from that of WT muscle fibers immediately after a bout of ECC contractions (Call et al., 2013). Skeletal muscle fibers are thought to be rendered inexcitable and contribute minimally to force development when depolarized above −55mV (Renaud and Light, 1992; Cairns et al., 1995, 1997). Although it is currently unknown how ECC contractions depolarize *mdx* muscle fibers (and likely mL172H and *Sgcb^−/−^* muscle fibers), putative mechanisms are loss of physical integrity of the plasmalemma as indicated by Evans Blue Dye (Lindsay et al., 2020), the development of branched fibers in dystrophic muscle (Chan et al., 2007; Head, 2010; Kiriaev et al., 2021), and/or dysfunction of ion channels (Yeung et al., 2005; Whitehead et al., 2006). For instance, it has been proposed that ECC contractions disrupt plasmalemmal ion channel function in branched fibers that results in ion flux, subsequently increasing intracellular Na^+^ and Ca^2+^ concentrations (Chan et al., 2007; Head, 2010). Future investigations will need to determine if the DGC (or lack thereof) results in branched fibers and if so, mechanistically how branched fibers contribute to NMJ transmission failure or the inability to generate and/or conduct an action potential along the plasmalemma.

While out of the scope of the present study, disruption downstream of the plasmalemma may also occur in dystrophic muscle due to ECC contractions. There is *ex vivo* evidence suggesting that function of the sarcoplasmic reticulum (SR) and myofibrillar apparatus are both involved in ECC-induced strength loss in *mdx* muscle (Bellinger et al., 2009; Blaauw et al., 2010; Morine et al., 2010; Lindsay et al., 2020). A caveat to *ex vivo* approaches is that plasmalemmal action potential generation and conduction is typically bypassed using these preps. Thus, SR dysfunction, loss of myofibrillar Ca^2+^ sensitivity, or structural damage to contractile proteins detected *ex vivo* in injured *mdx* muscle would not be detectable through *in vivo* measurements because loss of plasmalemmal excitability precedes them in the excitation-contraction coupling process.

In closing, we previously theorized that in order to attenuate ECC contraction-induced plasmalemmal inexcitability in *mdx* muscle, dystrophin would not only need to be present but also assembled into an intact and functional DGC (Baumann et al., 2020). Here, we confirm that even with WT levels of dystrophin in skeletal muscle, loss of plasmalemmal electrophysiological function will still occur during maximal ECC contractions if the DGC is otherwise disrupted (i.e., as in the *Sgcb^−/−^* mouse). Moreover, although a certain degree of protection is apparent in muscle with partially functional DGCs (i.e., the mL172H mouse), it remains susceptible to ECC contraction-induced plasmalemmal inexcitability. Our results highlight the significant role the DGC plays in maintaining plasmalemmal electrophysiological function and that a collective synergism (*via* each DGC protein) is required for this complex to function properly.

## Data Availability Statement

The raw data supporting the conclusions of this article will be made available by the authors, without undue reservation.

## Ethics Statement

The animal study was reviewed and approved by the Institutional Animal Care and Use Committees at the University of Minnesota.

## Author Contributions

CB, AL, GW, and DL conceived and designed the analysis. CB and SS collected the data and performed the analysis. CB, SS, JE, and DL contributed data or analysis tools. CB, AL, and SS wrote the paper. CB, AL, SS, JE, GW, and DL revised and approved the paper. All authors contributed to the article and approved the submitted version.

## Funding

This work was funded by the Research Endowment from the American College of Sports Medicine Foundation (to CB), a grant from the University of Minnesota Bob Allison Ataxia Research Center (to DL), and grants from the National Institutes of Health (T32-AG029796 and T32-AR007612 to CB and R01-AR042423 to JE).

## Conflict of Interest

The authors declare that the research was conducted in the absence of any commercial or financial relationships that could be construed as a potential conflict of interest.

## Publisher’s Note

All claims expressed in this article are solely those of the authors and do not necessarily represent those of their affiliated organizations, or those of the publisher, the editors and the reviewers. Any product that may be evaluated in this article, or claim that may be made by its manufacturer, is not guaranteed or endorsed by the publisher.

## References

[ref1] Araishi K. Sasaoka T. Imamura M. Noguchi S. Hama H. Wakabayashi E.. (1999). Loss of the sarcoglycan complex and sarcospan leads to muscular dystrophy in β-sarcoglycan-deficient mice. Hum. Mol. Genet. 8, 1589–1598. doi: 10.1093/hmg/8.9.1589, PMID: 10441321

[ref2] Basmajian J. V. De Luca C. J. (1985). Muscles Alive: Their Functions Revealed by Electromyography. Baltimore: Williams & Wilkins.

[ref3] Baumann C. W. Rogers R. G. Gahlot N. Ingalls C. P. (2014). Eccentric contractions disrupt FKBP12 content in mouse skeletal muscle. Physiol. Rep. 2:e12081. doi: 10.14814/phy2.12081, PMID: 25347864 PMC4187567

[ref4] Baumann C. W. Warren G. L. Lowe D. A. (2020). Plasmalemma function is rapidly restored in mdx muscle after eccentric contractions. Med. Sci. Sports Exerc. 52, 354–361. doi: 10.1249/MSS.0000000000002126, PMID: 31415447 PMC6962540

[ref5] Belanto J. J. Olthoff J. T. Mader T. L. Chamberlain C. M. Nelson D. M. McCourt P. M.. (2016). Independent variability of microtubule perturbations associated with dystrophinopathy. Hum. Mol. Genet. 25, 4951–4961. doi: 10.1093/hmg/ddw318, PMID: 28171583 PMC6078591

[ref6] Bell C. D. Conen P. E. (1968). Histopathological changes in Duchenne muscular dystrophy. J. Neurol. Sci. 7, 529–544. doi: 10.1016/0022-510X(68)90058-0, PMID: 5709861

[ref7] Bellinger A. M. Reiken S. Carlson C. Mongillo M. Liu X. Rothman L.. (2009). Hypernitrosylated ryanodine receptor calcium release channels are leaky in dystrophic muscle. Nat. Med. 15, 325–330. doi: 10.1038/nm.1916, PMID: 19198614 PMC2910579

[ref8] Blaauw B. Agatea L. Toniolo L. Canato M. Quarta M. Dyar K. A.. (2010). Eccentric contractions lead to myofibrillar dysfunction in muscular dystrophy. J. Appl. Physiol. 108, 105–111. doi: 10.1152/japplphysiol.00803.2009, PMID: 19910334

[ref9] Brooks S. V. (1998). Rapid recovery following contraction-induced injury to in situ skeletal muscles in mdx mice. J. Muscle Res. Cell Motil. 19, 179–187. doi: 10.1023/A:1005364713451, PMID: 9536444

[ref10] Cairns S. P. Flatman J. A. Clausen T. (1995). Relation between extracellular [K+], membrane potential and contraction in rat soleus muscle: modulation by the Na+-K+ pump. Pflugers Arch. 430, 909–915. doi: 10.1007/BF01837404, PMID: 8594543

[ref11] Cairns S. P. Hing W. A. Slack J. R. Mills R. G. Loiselle D. S. (1997). Different effects of raised [K+]o on membrane potential and contraction in mouse fast- and slow-twitch muscle. Am. J. Phys. 273, C598–C611. doi: 10.1152/ajpcell.1997.273.2.C598, PMID: 9277357

[ref12] Call J. A. Eckhoff M. D. Baltgalvis K. A. Warren G. L. Lowe D. A. (2011). Adaptive strength gains in dystrophic muscle exposed to repeated bouts of eccentric contraction. J. Appl. Physiol. 111, 1768–1777. doi: 10.1152/japplphysiol.00942.2011, PMID: 21960659 PMC3233886

[ref13] Call J. A. Warren G. L. Verma M. Lowe D. A. (2013). Acute failure of action potential conduction in *mdx* muscle reveals new mechanism of contraction-induced force loss. J. Physiol. 591, 3765–3776. doi: 10.1113/jphysiol.2013.254656, PMID: 23753524 PMC3752456

[ref14] Chan S. Head S. Morley J. (2007). Branched fibers in dystrophic mdx muscle are associated with a loss of force following lengthening contractions. Am. J. Physiol. Cell Physiol. 293, C985–C992. doi: 10.1152/AJPCELL.00128.2007, PMID: 17567750

[ref15] Constantin B. (2014). Dystrophin complex functions as a scaffold for signalling proteins. Biochim. Biophys. Acta 1838, 635–642. doi: 10.1016/j.bbamem.2013.08.023, PMID: 24021238

[ref16] Duddy W. Duguez S. Johnston H. Cohen T. Phadke A. Gordish-Dressman H.. (2015). Muscular dystrophy in the mdx mouse is a severe myopathy compounded by hypotrophy, hypertrophy and hyperplasia. Skelet. Muscle 5:16. doi: 10.1186/s13395-015-0041-y, PMID: 25987977 PMC4434871

[ref17] Durbeej M. Conn R. D. Hrstka R. F. Moore S. A. Allamand V. Davidson B. L.. (2000). Disruption of the β-sarcoglycan gene reveals pathogenetic complexity of limb-girdle muscular dystrophy type 2E. Mol. Cell 5, 141–151. doi: 10.1016/S1097-2765(00)80410-4, PMID: 10678176

[ref18] Garbincius J. F. Michele D. E. (2015). Dystrophin-glycoprotein complex regulates muscle nitric oxide production through mechanoregulation of AMPK signaling. Proc. Natl. Acad. Sci. U. S. A. 112, 13663–13668. doi: 10.1073/pnas.1512991112, PMID: 26483453 PMC4640723

[ref19] Head S. I. (2010). Branched fibres in old dystrophic mdx muscle are associated with mechanical weakening of the sarcolemma, abnormal Ca^2+^ transients and a breakdown of Ca^2+^ homeostasis during fatigue. Exp. Physiol. 95, 641–656. doi: 10.1113/expphysiol.2009.052019, PMID: 20139167

[ref20] Head S. Williams D. Stephenson D. (1992). Abnormalities in structure and function of limb skeletal muscle fibres of dystrophic mdx mice. Proceedings. Biol. Sci. 248, 163–169. doi: 10.1098/RSPB.1992.0058, PMID: 1352891

[ref21] Kinali M. Arechavala-Gomeza V. Cirak S. Glover A. Guglieri M. Feng L.. (2011). Muscle histology vs MRI in Duchenne muscular dystrophy. Neurology 76, 346–353. doi: 10.1212/WNL.0b013e318208811f, PMID: 21263136 PMC3034418

[ref22] Kiriaev L. Kueh S. Morley J. W. Houweling P. J. Chan S. North K. N.. (2021). Dystrophin-negative slow-twitch soleus muscles are not susceptible to eccentric contraction induced injury over the lifespan of the mdx mouse. Am. J. Physiol. Cell Physiol. 321, C704–C720. doi: 10.1152/ajpcell.00234.2021, PMID: 34432537

[ref23] Landisch R. Kosir A. Nelson S. Baltgalvis K. Lowe D. (2008). Adaptive and nonadaptive responses to voluntary wheel running by mdx mice. Muscle Nerve 38, 1290–1293. doi: 10.1002/mus.21141, PMID: 18816601 PMC3392332

[ref24] Lapidos K. A. Kakkar R. McNally E. M. (2004). The dystrophin glycoprotein complex: signaling strength and integrity for the sarcolemma. Circ. Res. 94, 1023–1031. doi: 10.1161/01.RES.0000126574.61061.25, PMID: 15117830

[ref25] Lindsay A. Baumann C. W. Rebbeck R. T. Yuen S. L. Southern W. M. Hodges J. S.. (2020). Mechanical factors tune the sensitivity of mdx muscle to eccentric strength loss and its protection by antioxidant and calcium modulators. Skelet. Muscle 10:3. doi: 10.1186/s13395-020-0221-2, PMID: 32007101 PMC6995146

[ref26] Lindsay A. Southern W. M. McCourt P. M. Larson A. A. Hodges J. S. Lowe D. A.. (2019). Variable cytoplasmic actin expression impacts the sensitivity of different dystrophin-deficient mdx skeletal muscle to eccentric contraction. FEBS J. 286, 2562–2576. doi: 10.1111/febs.14831, PMID: 30942954 PMC6613979

[ref27] Lowe D. A. Warren G. L. Ingalls C. P. Boorstein D. B. Armstrong R. B. (1995). Muscle function and protein metabolism after initiation of eccentric contraction-induced injury. J. Appl. Physiol. 79, 1260–1270. doi: 10.1152/jappl.1995.79.4.1260, PMID: 8567571

[ref28] Massopust R. Lee Y. Pritchard A. Nguyen V. McCreedy D. Thompson W. (2020). Lifetime analysis of mdx skeletal muscle reveals a progressive pathology that leads to myofiber loss. Sci. Rep. 10:17248. doi: 10.1038/s41598-020-74192-9, PMID: 33057110 PMC7560899

[ref29] Mázala D. Pratt S. Chen D. Molkentin J. Lovering R. Chin E. (2015). SERCA1 overexpression minimizes skeletal muscle damage in dystrophic mouse models. Am. J. Physiol. Cell Physiol. 308, C699–C709. doi: 10.1152/ajpcell.00341.2014, PMID: 25652448 PMC4420794

[ref30] McCourt J. L. Talsness D. M. Lindsay A. Arpke R. W. Chatterton P. D. Nelson D. M.. (2018). Mouse models of two missense mutations in actin-binding domain 1 of dystrophin associated with Duchenne or Becker muscular dystrophy. Hum. Mol. Genet. 27, 451–462. doi: 10.1093/hmg/ddx414, PMID: 29194514 PMC5886145

[ref31] Moens P. Baatsen P. H. Maréchal G. (1993). Increased susceptibility of EDL muscles from mdx mice to damage induced by contractions with stretch. J. Muscle Res. Cell Motil. 14, 446–451. doi: 10.1007/BF00121296, PMID: 7693747

[ref32] Morine K. Sleeper M. Barton E. Sweeney H. (2010). Overexpression of SERCA1a in the mdx diaphragm reduces susceptibility to contraction-induced damage. Hum. Gene Ther. 21, 1735–1739. doi: 10.1089/hum.2010.077, PMID: 20540606 PMC2999573

[ref33] Ng R. Banks G. B. Hall J. K. Muir L. A. Ramos J. N. Wicki J.. (2012). “Animal models of muscular dystrophy,” in Progress in Molecular Biology and Translational Science. ed. Conn P. M. (San Diego, CA: Elsevier B.V), 83–111. 10.1016/B978-0-12-394596-9.00004-4 PMC4872622 22137430

[ref34] Ohlendieck K. Campbell K. P. (1991). Dystrophin-associated proteins are greatly reduced in skeletal muscle from mdx mice. J. Cell Biol. 115, 1685–1694. doi: 10.1083/jcb.115.6.1685, PMID: 1757468 PMC2289197

[ref35] Pastoret C. Sebille A. (1995). mdx mice show progressive weakness and muscle deterioration with age. J. Neurol. Sci. 129, 97–105. doi: 10.1016/0022-510X(94)00276-T, PMID: 7608742

[ref36] Péladeau C. Adam N. Jasmin B. (2018). Celecoxib treatment improves muscle function in mdx mice and increases utrophin A expression. FASEB J. 32, 5090–5103. doi: 10.1096/fj.201800081R, PMID: 29723037

[ref37] Petrof B. J. Shrager J. B. Stedman H. H. Kelly A. M. Sweeney H. L. (1993). Dystrophin protects the sarcolemma from stresses developed during muscle contraction. Proc. Natl. Acad. Sci. U. S. A. 90, 3710–3714. doi: 10.1073/pnas.90.8.3710, PMID: 8475120 PMC46371

[ref38] Pratt S. J. P. Shah S. B. Ward C. W. Inacio M. P. Stains J. P. Lovering R. M. (2013). Effects of *in vivo* injury on the neuromuscular junction in healthy and dystrophic muscles. J. Physiol. 591, 559–570. doi: 10.1113/jphysiol.2012.241679, PMID: 23109110 PMC3577526

[ref39] Pratt S. J. P. Shah S. B. Ward C. W. Kerr J. P. Stains J. P. Lovering R. M. (2015). Recovery of altered neuromuscular junction morphology and muscle function in mdx mice after injury. Cell. Mol. Life Sci. 72, 153–164. doi: 10.1007/s00018-014-1663-7, PMID: 24947322 PMC4282693

[ref40] Renaud J. M. Light P. (1992). Effects of K+ on the twitch and tetanic contraction in the sartorius muscle of the frog, Rana pipiens. Implication for fatigue *in vivo*. Can. J. Physiol. Pharmacol. 70, 1236–1246. doi: 10.1139/y92-172, PMID: 1493591

[ref41] Roy P. Rau F. Ochala J. Messéant J. Fraysse B. Lainé J.. (2016). Dystrophin restoration therapy improves both the reduced excitability and the force drop induced by lengthening contractions in dystrophic mdx skeletal muscle. Skelet. Muscle 6, 23. doi: 10.1186/s13395-016-0096-4, PMID: 27441081 PMC4952281

[ref42] Rybakova I. N. Patel J. R. Ervasti J. M. (2000). The dystrophin complex forms a mechanically strong link between the sarcolemma and costameric actin. J. Cell Biol. 150, 1209–1214. doi: 10.1083/jcb.150.5.1209, PMID: 10974007 PMC2175263

[ref43] Schertzer J. Ryall J. Lynch G. (2006). Systemic administration of IGF-I enhances oxidative status and reduces contraction-induced injury in skeletal muscles of mdx dystrophic mice. Am. J. Physiol. Endocrinol. Metab. 291, E499–E505. doi: 10.1152/ajpendo.00101.2006, PMID: 16621899

[ref44] Sidky S. Ingalls C. Lowe D. Baumann C. (2021). Membrane proteins increase with the repeated bout effect. Med. Sci. Sports Exerc. doi: 10.1249/MSS.0000000000002762 [Epub ahead of print], PMID: 34334717 PMC8678180

[ref45] Straub V. Campbell K. P. (1997). Muscular dystrophies and the dystrophin-glycoprotein complex. Curr. Opin. Neurol. 10, 168–175. doi: 10.1097/00019052-199704000-00016, PMID: 9146999

[ref46] Vohra R. S. Lott D. Mathur S. Senesac C. Deol J. Germain S.. (2015). Magnetic resonance assessment of hypertrophic and pseudo-hypertrophic changes in lower leg muscles of boys with duchenne muscular dystrophy and their relationship to functional measurements. PLoS One 10:e0128915. doi: 10.1371/journal.pone.0128915, PMID: 26103164 PMC4477876

[ref47] Warren G. L. Hermann K. M. Ingalls C. P. Masselli M. R. Armstrong R. B. (2000). Decreased EMG median frequency during a second bout of eccentric contractions. Med. Sci. Sports Exerc. 32, 820–829. doi: 10.1097/00005768-200004000-00015, PMID: 10776902

[ref48] Warren G. L. Ingalls C. P. Armstrong R. B. (1998). A stimulating nerve cuff for chronic *in vivo* measurements of torque produced about the ankle in the mouse. J. Appl. Physiol. 84, 2171–2176. doi: 10.1152/jappl.1998.84.6.2171, PMID: 9609814

[ref49] Warren G. L. Ingalls C. P. Lowe D. A. Armstrong R. B. (2001). Excitation-contraction uncoupling: major role in contraction-induced muscle injury. Exerc. Sport Sci. Rev. 29, 82–87. doi: 10.1097/00003677-200104000-00008, PMID: 11337828

[ref50] Warren G. L. Ingalls C. P. Shah S. J. Armstrong R. B. (1999). Uncoupling of *in vivo* torque production from EMG in mouse muscles injured by eccentric contractions. J. Physiol. 515, 609–619. doi: 10.1111/j.1469-7793.1999.609ac.x, PMID: 10050026 PMC2269149

[ref51] Whitehead N. Streamer M. Lusambili L. Sachs F. Allen D. (2006). Streptomycin reduces stretch-induced membrane permeability in muscles from mdx mice. Neuromuscul. Disord. 16, 845–854. doi: 10.1016/j.nmd.2006.07.024, PMID: 17005404

[ref52] Yeung E. Whitehead N. Suchyna T. Gottlieb P. Sachs F. Allen D. (2005). Effects of stretch-activated channel blockers on [Ca2+]i and muscle damage in the mdx mouse. J. Physiol. 562, 367–380. doi: 10.1113/jphysiol.2004.075275, PMID: 15528244 PMC1665499

